# Impact of oral simvastatin therapy on acute lung injury in mice during pneumococcal pneumonia

**DOI:** 10.1186/1471-2180-12-73

**Published:** 2012-05-15

**Authors:** Angela R Boyd, Cecilia A Hinojosa, Perla J Rodriguez, Carlos J Orihuela

**Affiliations:** 1 Department of Microbiology and Immunology, The University of Texas Health Science Center at San Antonio, 7703 Floyd Curl Drive, San Antonio, TX, 78229-3900, USA

## Abstract

**Background:**

Recent studies suggest that the reported protective effects of statins (HMG-CoA reductase inhibitors) against community-acquired pneumonia (CAP) and sepsis in humans may be due to confounders and a healthy user-effect. To directly test whether statins are protective against *Streptococcus pneumoniae*, the leading cause of CAP, we examined the impact of prolonged oral simvastatin therapy at physiologically relevant doses in a mouse model of pneumococcal pneumonia. BALB/c mice were placed on rodent chow containing 0 mg/kg (control), 12 mg/kg (low simvastatin diet [LSD]; corresponds to 1.0 mg/kg/day), or 120 mg/kg (high simvastatin diet [HSD]; corresponds to 10 mg/kg/day) simvastatin for four weeks, infected intratracheally with *S. pneumoniae* serotype 4 strain TIGR4, and sacrificed at 24, 36, or 42 h post-infection for assessment of lung histology, cytokine production, vascular leakage and edema, bacterial burden and bloodstream dissemination. Some mice received ampicillin at 12-h intervals beginning at 48 h post-infection and were monitored for survival. Immunoblots of homogenized lung samples was used to assess ICAM-1 production.

**Results:**

Mice receiving HSD had reduced lung consolidation characterized by less macrophage and neutrophil infiltration and a significant reduction in the chemokines MCP-1 (*P* = 0.03) and KC (*P* = 0.02) and ICAM-1 in the lungs compared to control mice. HSD mice also had significantly lower bacterial titers in the blood at 36 (*P* = 0.007) and 42 (*P* = 0.03) hours post-infection versus controls. LSD had a more modest effect against *S. pneumoniae* but also resulted in reduced bacterial titers in the lungs and blood of mice after 42 h and a reduced number of infiltrated neutrophils. Neither LSD nor HSD mice had reduced mortality in a pneumonia model where mice received ampicillin 48 h after challenge.

**Conclusions:**

Prolonged oral simvastatin therapy had a strong dose-dependent effect on protection against *S. pneumoniae* as evidenced by reduced neutrophil infiltration, maintenance of vascular integrity, and lowered chemokine production in the lungs of mice on HSD. Statin therapy also protected through reduced bacterial burden in the lungs. Despite these protective correlates, mortality in the simvastatin-receiving cohorts was equivalent to controls. Thus, oral simvastatin at physiologically relevant doses only modestly protects against pneumococcal pneumonia.

## Background

Statins, or 3-hydroxy-3 methylglutaryl coenzyme A (HMG-CoA) reductase inhibitors, are prescribed to treat elevated levels of cholesterol and cardiovascular disease. As such they are among the most commonly prescribed drugs in the United States and worldwide. While statins can reduce plasma cholesterol by as much as 30-55%, statins also have potent anti-inflammatory and immunomodulatory properties that may be beneficial against certain infectious diseases in particular community-acquired pneumonia (CAP) [1].

In 2004, a prospective observational cohort study of individuals admitted to hospital for bacterial infection found that those taking statins had reduced incidence of sepsis and intensive care unit (ICU) admission [2]. Retrospective studies by Mortensen et al.*,* determined that prior statin use was associated with reduced 30-day mortality in patients admitted with CAP or sepsis [3,4]. Importantly, statin use was shown to reduce the risk of CAP in patients with diabetes, an established risk factor for CAP [5]. To date, greater than 20 independent studies have reported on the effects of statins on CAP and sepsis with a recent meta-analysis by Janda et al. reporting a strong beneficial effect against pneumonia- and sepsis-related mortality [6].

Importantly, not all studies identified a protective effect for statins against CAP [7-9]. For example a recent 2011 study by Yende et al., which accounted for healthy user effect and indication bias using propensity analysis, found no evidence for a protective effect in 1895 subjects hospitalized for CAP across 28 U.S. hospitals [9]. Likewise, in a study of 3415 individuals admitted to a hospital with pneumonia, Majundar et al. found that prior statin use had no effect on mortality or need for admission to an ICU [8]. Finally, de Saint Martin et al. found that statins users had higher ICU admission rates than non-users, albeit no differences in length of hospital stay or mortality were observed [7]. The authors of these studies suggest that the protective effects reported for statins may be due to confounders, a healthy user effect, and/or indication bias. As results from randomized control trials are not yet published, direct evidence of whether statins confer protection against CAP remains controversial.

Studies investigating the effects of statins on bacterial infections using laboratory animals have yielded conflicting results and added to the uncertainty. In a mouse model of *Klebsiella pneumoniae* pneumonia, lovastatin administration resulted in increased bacterial outgrowth that the authors attributed to reduced neutrophil accumulation within the lungs and defects in neutrophil-dependent intracellular killing [10]. For *Staphylococcus aureus*, high-dose statin therapy was shown to enhance the production of antimicrobial extracellular DNA traps by phagocytes within the lungs of mice and to protect against disseminated infection [11,12]. We have recently shown that short-term simvastatin therapy reduced the severity of pneumococcal disease in mice with sickle-cell disease but had no protective effect on young wild type mice [13]. Statin-mediated protection in the sickle-cell animals was due to: 1) reduced levels of Platelet-activating factor receptor, a host-protein that *Streptococcus pneumoniae* co-opts to adhere and invade host cells, and 2) reduced cytotoxicity of pneumolysin, a cholesterol dependent pore-forming toxin produced by *S. pneumoniae*. Of note, for all the animal studies described above, statins were either administered through a non-oral route, on a short-term basis, or at doses that far exceed what would normally be administered to humans for cardiovascular disease. Thus the mechanisms that might protect humans against pneumonia following oral statin therapy remain in question.

Given the large number of individuals at risk for pneumonia, it is important to determine whether prolonged oral statin therapy confers protection against pneumonia and if so the mechanisms that are responsible. For this reason we examined the effect of 4-week enteric-delivered simvastatin on the progression and severity of pneumococcal pneumonia in mice. Our findings indicate that simvastatin therapy reduced bacterial burden, decreased neutrophil influx, and diminished lung damage, but did not affect overall mortality. These studies provide experimental evidence supporting the notion that prophylactic statin therapy can exert protective benefits against CAP in humans; however these effects are modest in mice at the maximum recommended dose of simvastatin for humans.

## Materials and methods

### Mice and simvastatin diet

All experiments were performed in compliance with approved Institutional Animal Care and Use Committee protocols. Female 12-16 week old BALB/c mice were purchased from The Jackson Laboratory (Bar Harbor, MA). Rodent chow containing simvastatin (Sigma, St. Louis MO) at 0 mg/kg (control), 12 mg/kg (low simvastatin diet [LSD]), or 120 mg/kg (high simvastatin diet [HSD]) was prepared by Purina TestDiet (Richmond, IN) and fed *ad libitum* for ≥4 weeks. For a 25-30 g mouse consuming 2-2.5 g of chow per day these diets correspond to 1.0 and 10 mg/kg/day of simvastatin, respectively. Previous studies have confirmed a therapeutic effect for LSD and HSD by testing for a reduction in serum cholesterol [14].

### Assessment of disease severity

*S. pneumoniae* serotype 4, strain TIGR4 was grown in Todd Hewitt Broth at 37°C in 5% CO_2_[15]. Animals were anesthetized with vaporized isoflurane and 10^5^ cfu in 100 μl phosphate-buffered saline (PBS) was delivered intratracheally by forced inhalation [16]. Mice were euthanized and bacterial burden in the lungs was assessed per gram of homogenized tissue. Alternatively, bacteremia and mortality was assessed over 7 days [17]. In intervention experiments, beginning at 48 h post-challenge, mice were administered ampicillin (80 mg/kg) at 12 h intervals. Lungs sections (5 μm) were stained with Hematoxylin and Eosin (H&E) and scored in a blind manner based on lung consolidation, evidence of hemorrhage, and extent of cellular infiltration.

### Bronchoalveolar lavage (BAL)

Mice were euthanized by CO_2_ asphyxiation. Following surgical visualization of the trachea, BAL was performed by insertion of a 0.18 gauge angiocatheter and flushing of the lungs with 0.5 ml ice-cold PBS until a total volume of 3 ml was obtained. BAL fluid was strained (40-μM) and centrifuged. The cellular fraction was suspended in 1 ml PBS and total cell counts were determined using a hemocytometer. Differential cell counts were done following cytospin and staining with a Diff-Quick Staining Kit (IMEB Inc.); >300 cells were counted in three separate fields for each mouse.

### Albumin and cytokine analysis

Vascular leakage in BAL fluid was assessed using a mouse albumin ELISA Quantitation Set (Bethyl Laboratories, Inc., Montgomery, TX). Levels of Tumor Necrosis Factor (TNF)α, Interleukin (IL)-6, IL-10, IL-12, Monocyte chemoattractant protein (MCP)-1, and Interferon (IFN)γ in BAL fluid and serum samples were performed using a Mouse Inflammatory Cytometric Bead Array (BD Biosciences). Concentrations of KC were determined using a DuoSET Mouse CXCL1/KC ELISA kit from R&D Systems (Minneapolis, MN).

### Determination of ICAM-1 protein levels in the lungs

Lungs were homogenized in RIPA buffer containing a protease inhibitor cocktail (Sigma). Separation of protein by SDS-PAGE, transfer to nitrocellulose membrane, and detection was performed using standard immunoblot methods. Goat polyclonal antibody to ICAM-1 (Santa Cruz Biotechnology) was used for detection. Relative protein levels were determined by densitometric analysis of Western blot bands using a Molecular Imager Gel Doc XR System (BioRad, Hercules, CA). To ensure that equal amount of protein had been probed, and to permit normalization of ICAM-1 across samples, membranes were stripped and the amount of actin determined using rabbit anti-actin antibodies (Bethyl Laboratories, Inc., Montgomery, TX).

### Statistical analysis

For comparisons between cohorts either a One-way ANOVA or two-tailed Student’s *t* test was used as indicated. *P* values <0.05 were considered significant. For survival analyses a Kaplan-Meier Log Rank Survival Test was used.

## Results

### Oral statin prophylaxis decreases the severity of pneumococcal pneumonia in mice

**Figure 1:**
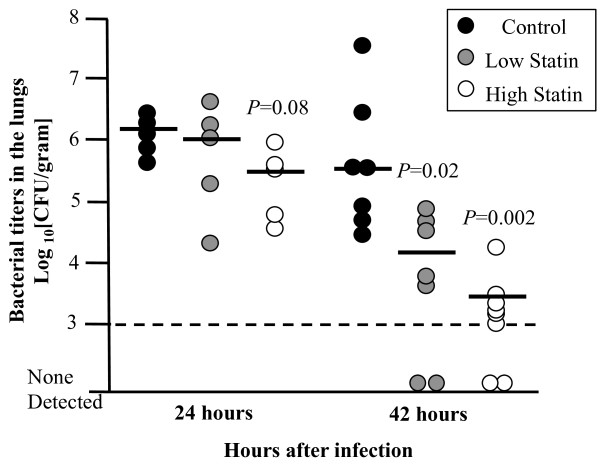
**Simvastatin prophylaxis decreases bacterial burdens in the lungs.** Bacterial titers in the lungs 24 and 42 h after infection of mice fed the Control, Low or High statin diet and challenged intratracheally with 1 X 10^5^ cfu. Each circle represents an individual mouse. Horizontal lines indicate the median; dashed lines indicate limit of detection Mice receiving statins had significantly lower bacterial titers in the lungs 42 h after infection. Data are presented as the mean ± SEM. Statistics were determined by a two-tailed student’s *t*-test. *P* < 0.05 was considered significant on comparison to Control fed mice.

**Figure 2:**
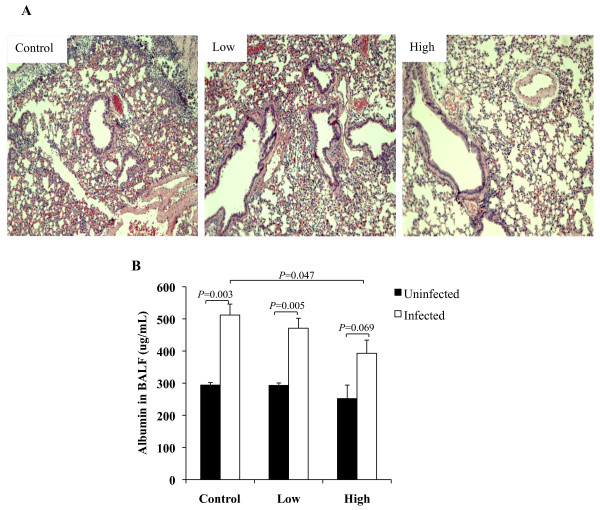
**Simvastatin prophylaxis decreases histopathology and vascular leakage.A**) Representative micrographs of Hematoxylin- and Eosin-stained lung sections from mice 42 h after infection. Note that the high statin fed mice exhibit reduced cellularity and vascular hemorrhage. Original magnification, 10X. **B**) Vascular integrity was determined by assessing the amount of albumin present in the BAL fluid by ELISA prior to and following infection (n = 3/group for uninfected and n = 6/group for infected mice). Data are presented as the mean ± SEM. Statistics were determined by a two-tailed student’s *t*-test. *P* < 0.05 was considered significant in comparison to Control fed mice.

To determine the effect of simvastatin prophylaxis on disease severity we first assessed bacterial burden during pneumonia. Pneumococcal titers in the lungs collected at 24 h post-infection (hpi) did not significantly differ between the simvastatin fed and control cohorts (Figure 1); although bacterial titers in the lungs of mice on HSD had a trend towards reduced bacterial load (*P* = 0.08). At 42 hpi, mice on the control diet had approximately 50- (*P =* 0.02) and 100-fold (*P* = 0.002) more bacteria in their lungs than mice on LSD and HSD, respectively. In agreement with this reduced bacterial load, histological analysis of lung sections demonstrated decreased lung damage with less evidence of lung consolidation, edema, and hemorrhage in the HSD mice versus controls (Figure 2A). Mice receiving LSD had no discernible difference in lung damage versus controls. Analysis of BAL fluid for evidence of vascular leakage demonstrated that mice on HSD had reduced albumin in their lungs 24 hpi (Figure 2B). No differences in albumin levels were found between mice receiving the LSD versus the control diet or in baseline levels of albumin prior to infection. Thus, HSD seemed to protect vascular integrity during infection.

**Figure 3:**
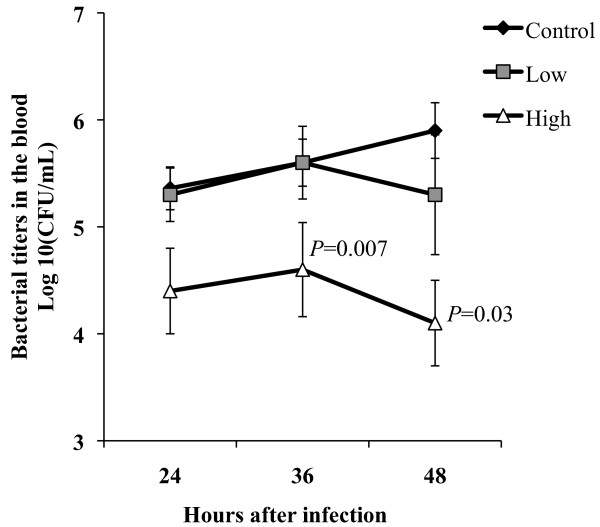
**Mice on simvastatin prophylaxis show enhanced protection from bacteremia.** Bacterial titers in the blood of challenged mice 24, 36 and 42 h after infection. Mice on Control (n = 11), Low (n = 11) or High (n = 12) diet were challenged intratracheally with 1 X 10^5^ cfu. Mice receiving statins had significantly fewer bacteria in the blood. Data are presented as the mean ± SEM. Statistics were determined by a two-tailed student’s *t*-test. *P* < 0.05 was considered significant.

We subsequently examined the impact of oral simvastatin therapy on development of bacteremia. Following intratracheal challenge at 24 hpi, bacterial titers in the blood were not significantly different among all three groups tested; although mice receiving HSD had lower median titers compared to mice on the control diet (*P* = 0.12) (Figure 3). Between 24 and 36 h, pneumococcal titers in the blood increased at a similar rate for all mice, nonetheless mice on HSD had significantly fewer pneumococci in their blood compared to control mice (*P* = 0.007). After 36 h, mice receiving the control diet continued to experience bacterial replication whereas those on a simvastatin diet maintained or began to clear bacteria from the blood. At 42 hpi, mice on the HSD continued to have significantly less bacterial titers in the blood compared to control fed mice (*P* = 0.03).

### High-dose simvastatin reduces chemokine production in the lungs

**Figure 4:**
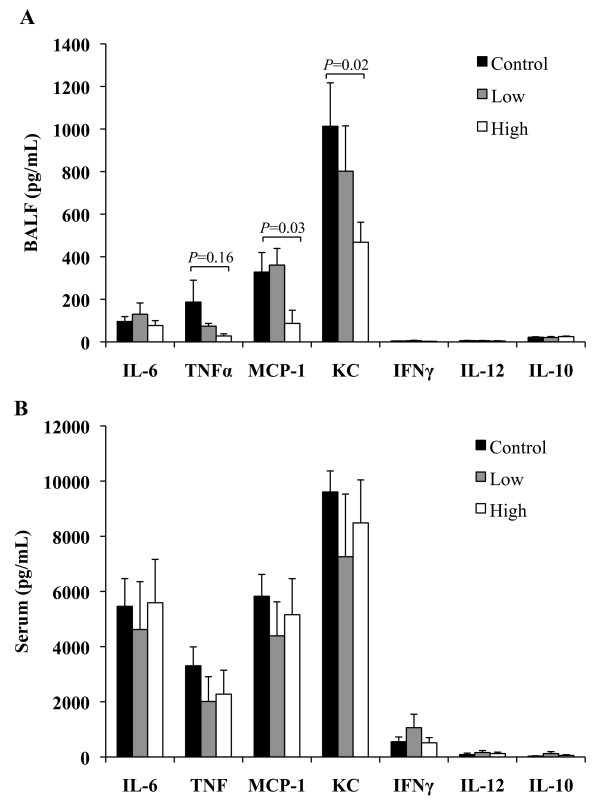
**Statins preferentially decrease chemokine production in the lungs without reducing proinflammatory mediators during early pneumococcal pneumonia.** Control, Low, and High statin mice were challenged intratracheally with 1 X 10^5^ cfu and sacrificed 24 h after infection. Collected **A**) bronchoalveolar lavage fluid and **B**) serum were assayed for pro-inflammatory cytokine and chemokine production by a mouse inflammatory cytometric bead array or ELISA (n = 12/group). No statistically significant differences in cytokine production were observed, while the chemokines MCP-1 and KC were significantly decreased in mice receiving the high statin diet compared to control. Data are presented as the mean ± SEM. Statistics were determined by a two-tailed student’s *t*-test. *P* < 0.05 was considered significant in comparison to Control fed mice.

Statins have been reported to reduce cytokine production following LPS stimulation of monocytes and decrease lung inflammation following instillation of LPS in healthy human volunteers [18,19]. Thus we investigated the effect of simvastatin therapy on the local and systemic production of cytokines and chemokines during pneumococcal pneumonia. At 24 hpi, before bacterial titers in the lungs were significantly different, no differences were observed for TNFα, IL-6, IL-10, IL-12, MCP-1, KC and IFNγ in the BAL fluid or serum of mice on LSD versus controls (Figure 4A, B). In contrast, mice on HSD had significant reductions in MCP-1 (*P* = 0.03) and KC (*P* = 0.02) in the BAL fluid but not serum. No differences were observed for all other cytokines or chemokines in the BALF or in the serum of HSD mice. Uninfected mice on control, LSD and HSD had unaltered low levels of cytokines and chemokines present in the BAL fluid and serum prior to infection (data not shown).

### Statins impact neutrophil influx and ICAM-1 expression

**Figure 5:**
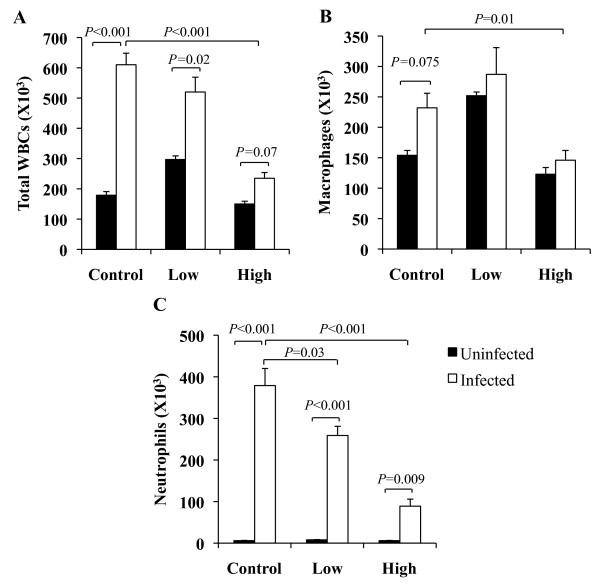
**Statins decrease leukocyte infiltration into the lungs.A**) Total cell counts obtained by bronchoalveolar lavage (BAL) 24 h after intratracheal infection with 1 X 10^5^ cfu were determined by visual counting using a hemocytometer (n = 6/group). Differential cell counts of cytospins prepared from the same BAL demonstrating **B**) lower monocytes/macrophages in mice receiving the high statin diet and C) a dose-dependent reduction in neutrophil influx 24 h after infection. Data are presented as the mean ± SEM. Statistics were determined by a two-tailed student’s *t*-test. *P* < 0.05 was considered significant in comparison to Control fed mice.

Statins have been reported to reduce neutrophil influx into the lungs following instillation of LPS and during *K. pneumoniae* infection [10]. We therefore assessed whether oral simvastatin also attenuated cellular influx into the lungs during pneumococcal pneumonia. Total cell counts using BAL fluid collected at 24 hpi demonstrated that mice receiving HSD had significantly less cellular infiltration compared to control mice (*P* < 0.001) (Figure 5A). Notably, infected HSD mice had only a nominal increase in cellular infiltrates (*P* = 0.07 versus controls) versus the mock-infected controls, confirming that high-dose statins indeed reduced leukocyte influx. In contrast, mice on control and LSD had a robust and significant cellular response versus uninfected controls (Control, *P* < 0.001; LSD, *P* = 0.02).

Although during infection the absolute numbers of leukocytes in the BAL did not differ between mice on LSD and control diet, those receiving LSD had significantly less neutrophils in the BAL compared to control fed mice (*P* = 0.03) (Figure 5C). Mice receiving HSD also had a significant reduction in the number of infiltrating neutrophils (*P* < 0.001). Differences in neutrophil numbers were dose-dependent with those on the LSD and HSD at approximately 75% and 25% of the levels observed for the control diet, respectively. Importantly, a less dramatic effect was observed for macrophages/monocytes. Only infected mice on HSD had a significant reduction in numbers versus their infected controls (*P* = 0.01) (Figure 5B).

**Figure 6:**
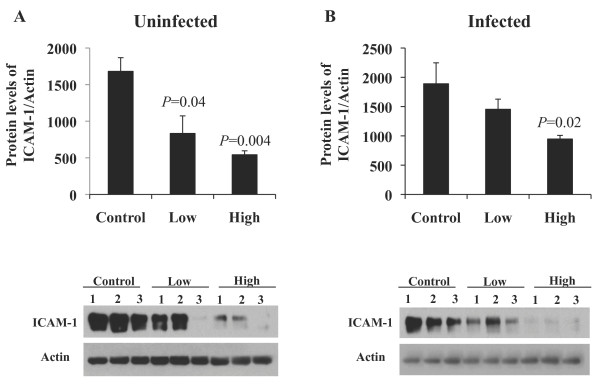
**Statins decrease ICAM-1 protein expression prior to and following infection with*S. pneumoniae.*** Lungs from mice on Control, Low, and High statin diet (n = 6/group) were examined for protein expression of ICAM-1 prior to and 24 h following intratracheally infection with 1 X 10^5^ cfu by western blot analysis of whole lung protein lysates (n = 3/group for uninfected and n = 6/group for infected mice). Mice receiving statins had significantly less ICAM-1 protein levels present in the lungs both **A**) prior to and **B**) following infection. Data are presented as the mean ± SEM. Statistics were determined by a two-tailed student’s *t*-test. *P* < 0.05 was considered significant in comparison to Control fed mice.

Statins have been found to decrease the up regulation of adhesion molecules on endothelial cells in several models of inflammation [20-22]. Because we observed a dose-dependent reduction in neutrophil influx, yet only mice on the HSD had lower production of the neutrophil chemoattractant KC, we went on to assess whether statins were reducing neutrophil infiltration by modulating the upregulation of adhesion molecules. In agreement with the observed reduction in neutrophils influx, mice receiving statins had a strong dose-dependent reduction in the protein levels of ICAM-1 present in the lungs prior to infection with *S. pneumoniae* (Control versus LSD, *P* = 0.04; Control versus HSD, *P* = 0.004) (Figure 6A). Whereas at 24 hpi, only mice on the HSD continued to have decreased protein levels of ICAM-1 in the lungs (*P* = 0.02) (Figure 6B). Taken together these findings suggest that statins exert a dose-dependent effect to reduce neutrophil infiltration during pneumococcal pneumonia by reducing neutrophil chemotaxis and transcytosis without suppressing pro-inflammatory mediators required to enhance antibacterial defense mechanism.

#### Surivival of infected mice on statins

**Figure 7:**
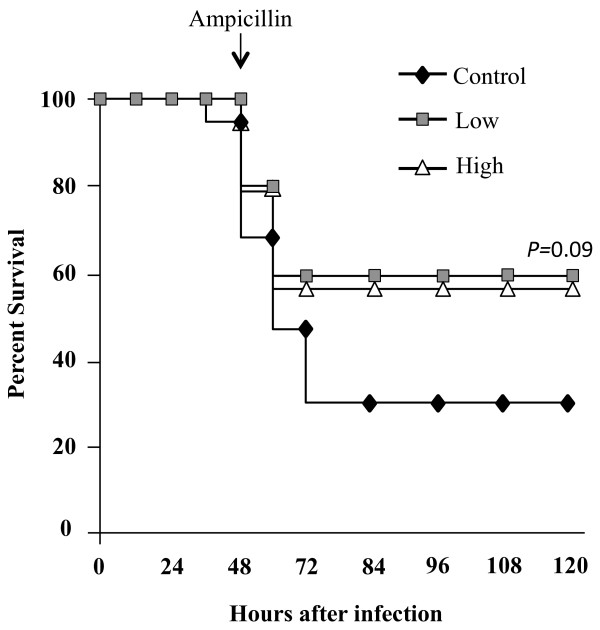
**Survival of simvastatin fed mice following infection with*S. pneumoniae*.** Kaplan–Meier plot demonstrating percent survival of challenged mice. Mice on Control (n = 19), Low (n = 19) or High (n = 20) diet were challenged intratracheally with 1 X 10^5^ cfu. After 48 h ampicillin (80 mg/kg) was administered every twelve hours. Significance was determined by Log-Rank test.

Finally, we sought to directly test if prophylactic statin therapy improved disease outcomes in a clinically relevant infection model. Since individuals with CAP receive antimicrobials, we tested for survival of mice in a model where beginning at 48 hpi mice received ampicillin at 12 h intervals. Despite the protective effects observed above, mice on LSD or HSD had equivalent survival over time as controls (Figure 7). Thus, the overall protective effects of statins were modest and may not necessarily impact disease outcomes in humans.

## Discussion

Numerous studies, including recent meta-analyses, have reported an association between prior statin use and improved outcomes following CAP and sepsis [6,23]. Conversely, a recent study by Yende et al., in which the authors accounted for healthy user effect and indication bias using propensity analysis, found no evidence for a protective effect of statins on clinical outcomes [9]. Experiments with laboratory animals can directly test whether statins are protective against infectious diseases. Unfortunately, studies thus far have been flawed by either short-duration of statin therapy, the administration of too high levels of statins, and non-enteric delivery of the drug. Thus, at the onset of our study, it was unclear whether prolonged simvastatin therapy, delivered enterically for 4 weeks, and at a dose taken by humans for high cholesterol (LSD; 1.0 mg/kg/day) would be protective against *S. pneumoniae*. Most evident from our studies was the strong dose-dependent effect of simvastatin on examined parameters; for LSD we observed only reduced bacterial titers in the lungs at 42 hpi and intermediate reductions in neutrophil influx and ICAM-1 expression. In contrast, mice receiving HSD (i.e. 10 mg/kg/day; 10X maximum recommended human dose) had significantly reduced bacterial titers in the lungs and blood, less lung damage, and diminished cytokine production and neutrophil recruitment. Therefore, the dose of statin received by participating individuals in human trials for statins should be a key consideration.

Excessive cellular infiltrates is a common complication of CAP. Likewise, ours is not the first study to suggest that statins attenuate neutrophil influx or edema during infection or injury [12,19,24-26]; with a similar effect for lovastatin (10 mg/kg administered intraperitoneal 20, 12, and 0.5 h preceding exposure) reported by Fessler et al. during *K. pneumoniae* infection [10]. Based on our findings, reduced neutrophil infiltration can now be attributed to reduced chemokine production (e.g. MCP-1 and KC) as well as a reduction in ICAM-1 levels that together reduces the entry of circulating neutrophils and macrophages into the lungs. Notably, and despite considerable lung protection observed in HSD mice, we found no significant differences in IL-6 or TNFα levels, the two pro-inflammatory cytokines most often correlated with severity of CAP [27]. Thus, in our study simvastatin did not prevent a “cytokine storm” and instead acted through decreased cellular infiltration and vascular leakage. This cautions against using IL-6 and TNFα as correlates of protection in human trials. Of note, this contrasts findings by Ando et al., where pretreatment of the mice with cerivastatin (20 mg/kg *i.p*. 12 and 1 h before challenge) reduced serum levels of TNFα and IL-1β following LPS administration (15 mg/kg *i.p.*). This difference may be due to the use of different statins, distinct routes of drug administration and bacterial challenge, our use of a lower dose, and because we examined the response to a Gram-positive bacteria [26]. The latter applies to all the aforementioned animal studies as well. It is worth noting that statins have a well-documented anti-inflammatory effect that is independent of infection. For example, Müller et al., found that simvastatin treatment limited pulmonary endothelial injury, attenuated pulmonary hyperpermeability, prevented the recruitment of leukocytes to the lung, reduced pulmonary cytokine levels and improved oxygenation in mechanically ventilated mice [28]. Thus our findings for HSD are consistent with those of Müller et al.

During pneumonia, neutrophils are the primary effector cell responsible for clearance of extracellular bacteria. It was therefore paradoxical that reduced neutrophil infiltration was observed in HSD mice simultaneously to decreased bacterial burden in their lungs. In our hands, simvastatin does not have antibacterial effects *in vitro* on *S. pneumoniae* at *in vivo* concentrations [13,29]. Yet, Jerwood et al. have shown that simvastatin has considerable antimicrobial properties against *Staphylococcus aureus*[30]. Thus we cannot directly rule out killing by simvastatin *in vivo*. Possible reasons for the reduction in bacterial burden also include lowered PAFr expression in the lungs that would decrease bacterial adhesion and/or enhanced killing ability by resident alveolar macrophages due to enhanced resistance to the cholesterol-dependent toxin pneumolysin [13]. Although not tested in our study, high dose statins has also been reported to increase killing of *S. aureus* and *S. pneumoniae* by enhancing the formation of phagocyte extracellular traps in mice fed pulverized rodent chow supplemented with 500 mg/kg simvastatin [11]. For *S. pneumoniae,* killing by extracellular traps remains controversial as other investigators have shown that *S. pneumoniae* is able to resist neutrophil extracellular traps (NETs) due to the presence of a surface localized endonuclease that degrades the DNA scaffold of NETs [31,32].

Importantly, the discrepancy in disease severity in mice for *S. pneumoniae* with simvastatin and for *K. pneumoniae* with lovastatin, as reported by Fessler et al. [10], raises the possibility that statins facilitate differential outcomes depending on the infectious agent. Neutrophils are a primary mediator of lung injury during pneumonia and a study with neutropenic mice infected with *S. pneumoniae* demonstrated less lung injury and improved survival [33,34]. Our findings are consistent with these previous publications. In addition to the differences in the class and delivery of statins used between our study and that of Fessler et al., another important consideration is that Gram-negative bacteria do not produce cholesterol-dependent cytolysins, such as pneumolysin. Statins might preferentially protect against Gram-positive bacteria. Such species-specific effects would have considerable implications regarding the utility of statins as a prophylactic drug within a hospital setting where the etiological cause of pneumonia is more frequently Gram-negative bacteria [35]. This further suggests that statin studies consider the etiological agent responsible for CAP.

In our clinical intervention model we observed no protection for challenged LSD or HSD mice as measured by survival. Worth consideration is the possibility that improved survival might have been observed if the ampicillin therapy was begun earlier. This notion is supported by the reduced bacterial titers in the blood of mice on LSD and HSD at 42 h hpi. Nonetheless, this observation suggests that the protective effects of statins are overall modest; even when a high dose of simvastatin is administered. One important caveat is that rodents metabolize drugs at different rates than humans and so the protective effect of statins may be more pronounced in humans at lower doses. Nonetheless our findings strongly suggest that individuals taking lower levels of statins would have reduced-protection against CAP versus those on higher doses. Finally, these observations highlight the complexity of the clinical question and indicate that human trials on statins for CAP should have multiple correlates of protection.

## Conclusion

In summary, this study demonstrates that oral statin therapy may protect against pneumococcal pneumonia by increasing bacterial clearance, reducing excessive neutrophil infiltration, and decreasing vascular permeability. Importantly, a strong dose-dependent effect was observed for simvastatin with minimal effects on mice administered the maximum recommended human dose (i.e. LSD) and no differences in overall survival observed for mice on HSD. Our observations help to explain why some human studies fail to find a protective effect as multiple correlates of protection are required for testing and multiple variables most likely affect outcomes including statin dose, etiological cause of CAP, and severity of CAP at time of admission.

## Author contributions

ARB, CH, and PJR performed the experiments and generated the data. ARB and CJO contributed to the conception and design of the experiments performed as well as the writing of the manuscript. All authors read and approved the final manuscript.

## References

[B1] Corrales-Medina VF Musher DM Immunomodulatory agents in the treatment of community-acquired pneumonia: a systematic review J Infect 2011 63 3 187 199 10.1016/j.jinf.2011.06.009 21763343

[B2] Almog Y Shefer A Novack V Maimon N Barski L Eizinger M Friger M Zeller L Danon A Prior statin therapy is associated with a decreased rate of severe sepsis Circulation 2004 110 7 880 885 10.1161/01.CIR.0000138932.17956.F1 15289367

[B3] Mortensen EM Restrepo MI Anzueto A Pugh J The effect of prior statin use on 30-day mortality for patients hospitalized with community-acquired pneumonia Respir Res 2005 6 82 10.1186/1465-9921-6-82 16042797 PMC1199623

[B4] Mortensen EM Restrepo MI Copeland LA Pugh JA Anzueto A Cornell JE Pugh MJ Impact of previous statin and angiotensin II receptor blocker use on mortality in patients hospitalized with sepsis Pharmacotherapy 2007 27 12 1619 1626 10.1592/phco.27.12.1619 18041882

[B5] van de Garde EM Hak E Souverein PC Hoes AWvan den Bosch JM 2006 Statin therapy and reduced risk of pneumonia in patients with diabetes. Thorax, Leufkens HG 10.1136/thx.2006.062885 PMC2121156 16809409

[B6] Janda S Young A Fitzgerald JM Etminan M Swiston J The effect of statins on mortality from severe infections and sepsis: a systematic review and meta-analysis J Crit Care 2010 25 4 e657 622 656 10.1016/j.jcrc.2010.02.013 20413251

[B7] De Saint Martin L Tande D Goetghebeur D Pan-Lamande M Segalen Y Pasquier E Statin use does not affect the outcome of acute infection: a prospective cohort study Presse Med 2010 39 3 e52 57 10.1016/j.lpm.2009.09.022 20022215

[B8] Majumdar SR McAlister FA Eurich DT Padwal RS Marrie TJ Statins and outcomes in patients admitted to hospital with community acquired pneumonia: population based prospective cohort study BMJ 2006 333 7576 999 10.1136/bmj.38992.565972.7C 17060337 PMC1635620

[B9] Yende S Milbrandt EB Kellum JA Kong L Delude RL Weissfeld LA Angus DC Understanding the potential role of statins in pneumonia and sepsis Crit Care Med 2011 39 8 1871 1878 10.1097/CCM.0b013e31821b8290 21516038 PMC3139804

[B10] Fessler MB Young SK Jeyaseelan S Lieber JG Arndt PG Nick JA Worthen GS A role for hydroxy-methylglutaryl coenzyme a reductase in pulmonary inflammation and host defense Am J Respir Crit Care Med 2005 171 6 606 615 15591471 10.1164/rccm.200406-729OC

[B11] Chow OA von Kockritz-Blickwede M Bright AT Hensler ME Zinkernagel AS Cogen AL Gallo RL Monestier M Wang Y Glass CK Statins enhance formation of phagocyte extracellular traps Cell Host Microbe 2010 8 5 445 454 10.1016/j.chom.2010.10.005 21075355 PMC3008410

[B12] McDowell SA Ma Y Kusano R 2011 Simvastatin is Protective During Staphylococcus aureus Pneumonia. Curr Pharm Biotechnol, Akinbi HT 10.2174/138920111798281027 PMC4469268 21401521

[B13] Rosch JW Boyd AR Hinojosa E Pestina T Hu Y Persons DA Orihuela CJ Tuomanen EI Statins protect against fulminant pneumococcal infection and cytolysin toxicity in a mouse model of sickle cell disease J Clin Invest 2010 120 2 627 635 10.1172/JCI39843 20093777 PMC2810080

[B14] Miller RA Harrison DE Astle CM Baur JA Boyd AR de Cabo R Fernandez E Flurkey K Javors MA Nelson JF Rapamycin, but not resveratrol or simvastatin, extends life span of genetically heterogeneous mice J Gerontol A Biol Sci Med Sci 2011 66 2 191 201 20974732 10.1093/gerona/glq178 PMC3021372

[B15] Tettelin H Nelson KE Paulsen IT Eisen JA Read TD Peterson S Heidelberg J DeBoy RT Haft DH Dodson RJ Complete genome sequence of a virulent isolate of Streptococcus pneumoniae Science 2001 293 5529 498 506 10.1126/science.1061217 11463916

[B16] Hinojosa E Boyd AR Orihuela CJ Age-associated inflammation and toll-like receptor dysfunction prime the lungs for pneumococcal pneumonia J Infect Dis 2009 200 4 546 554 10.1086/600870 19586419 PMC3102250

[B17] Orihuela CJ Gao G Francis KP Yu J Tuomanen EI Tissue-specific contributions of pneumococcal virulence factors to pathogenesis J Infect Dis 2004 190 9 1661 1669 10.1086/424596 15478073

[B18] Methe H Kim JO Kofler S Nabauer M Weis M Statins decrease Toll-like receptor 4 expression and downstream signaling in human CD14+ monocytes Arterioscler Thromb Vasc Biol 2005 25 7 1439 1445 10.1161/01.ATV.0000168410.44722.86 15860745

[B19] Shyamsundar M McKeown ST O'Kane CM Craig TR Brown V Thickett DR Matthay MA Taggart CC Backman JT Elborn JS Simvastatin decreases lipopolysaccharide-induced pulmonary inflammation in healthy volunteers Am J Respir Crit Care Med 2009 179 12 1107 1114 10.1164/rccm.200810-1584OC 19324974 PMC2695496

[B20] Takeuchi S Kawashima S Rikitake Y Ueyama T Inoue N Hirata K Yokoyama M Cerivastatin suppresses lipopolysaccharide-induced ICAM-1 expression through inhibition of Rho GTPase in BAEC Biochem Biophys Res Commun 2000 269 1 97 102 10.1006/bbrc.2000.2238 10694484

[B21] Okouchi M Okayama N Omi H Imaeda K Shimizu M Fukutomi T Itoh M Cerivastatin ameliorates high insulin-enhanced neutrophil-endothelial cell adhesion and endothelial intercellular adhesion molecule-1 expression by inhibiting mitogen-activated protein kinase activation J Diabetes Complications 2003 17 6 380 386 10.1016/S1056-8727(02)00245-3 14583185

[B22] Kimura M Kurose I Russell J Granger DN Effects of fluvastatin on leukocyte-endothelial cell adhesion in hypercholesterolemic rats Arterioscler Thromb Vasc Biol 1997 17 8 1521 1526 10.1161/01.ATV.17.8.1521 9301630

[B23] Tleyjeh IM Kashour T Hakim FA Zimmerman VA Erwin PJ Sutton AJ Ibrahim T Statins for the prevention and treatment of infections: a systematic review and meta-analysis Arch Intern Med 2009 169 18 1658 1667 10.1001/archinternmed.2009.286 19822822

[B24] Yao HW Mao LG Zhu JP Protective effects of pravastatin in murine lipopolysaccharide-induced acute lung injury Clin Exp Pharmacol Physiol 2006 33 9 793 797 10.1111/j.1440-1681.2006.04440.x 16922808

[B25] Pirat A Zeyneloglu P Aldemir D Yucel M Ozen O Candan S Arslan G Pretreatment with simvastatin reduces lung injury related to intestinal ischemia-reperfusion in rats Anesth Analg 2006 102 1 225 232 10.1213/01.ane.0000189554.41095.98 16368834

[B26] Ando H Takamura T Ota T Nagai Y Kobayashi K Cerivastatin improves survival of mice with lipopolysaccharide-induced sepsis J Pharmacol Exp Ther 2000 294 3 1043 1046 10945857

[B27] Antunes G Evans SA Lordan JL Frew AJ Systemic cytokine levels in community-acquired pneumonia and their association with disease severity Eur Respir J 2002 20 4 990 995 10.1183/09031936.02.00295102 12412694

[B28] Muller HC Hellwig K Rosseau S Tschernig T Schmiedl A Gutbier B Schmeck B Hippenstiel S Peters H Morawietz L Simvastatin attenuates ventilator-induced lung injury in mice Crit Care 2010 14 4 R143 10.1186/cc9209 20673352 PMC2945124

[B29] Bergman P Linde C Putsep K Pohanka A Normark S Henriques-Normark B Andersson J Bjorkhem-Bergman L Studies on the antibacterial effects of statins–in vitro and in vivo PLoS One 2011 6 8 e24394 10.1371/journal.pone.0024394 21912631 PMC3166163

[B30] Jerwood S Cohen J Unexpected antimicrobial effect of statins J Antimicrob Chemother 2008 61 2 362 364 18086693 10.1093/jac/dkm496

[B31] Wartha F Beiter K Albiger B Fernebro J Zychlinsky A Normark S Henriques-Normark B Capsule and D-alanylated lipoteichoic acids protect Streptococcus pneumoniae against neutrophil extracellular traps Cell Microbiol 2007 9 5 1162 1171 10.1111/j.1462-5822.2006.00857.x 17217430

[B32] Beiter K Wartha F Albiger B Normark S Zychlinsky A Henriques-Normark B An endonuclease allows Streptococcus pneumoniae to escape from neutrophil extracellular traps Curr Biol 2006 16 4 401 407 10.1016/j.cub.2006.01.056 16488875

[B33] Abraham E Neutrophils and acute lung injury Crit Care Med 2003 31 4 Suppl S195 199 12682440 10.1097/01.CCM.0000057843.47705.E8

[B34] Marks M Burns T Abadi M Seyoum B Thornton J Tuomanen E Pirofski LA Influence of neutropenia on the course of serotype 8 pneumococcal pneumonia in mice Infect Immun 2007 75 4 1586 1597 10.1128/IAI.01579-06 17296760 PMC1865693

[B35] Lynch JP Hospital-acquired pneumonia: risk factors, microbiology, and treatment Chest 2001 119 2 Suppl 373S 384S 11171773 10.1378/chest.119.2_suppl.373s

